# Follow-up of vascular-targeted photodynamic therapy in a real-world setting

**DOI:** 10.1007/s00345-023-04738-9

**Published:** 2024-01-20

**Authors:** Angelika Borkowetz, Jeremy Kwe, Katharina Boehm, Martin Baunacke, Roman Herout, Marius Lucke, Adriana Burcea, Christian Thomas

**Affiliations:** 1 Department of Urology, University Hospital Carl Gustav Carus, Technische Universität Dresden, Fetscherstraße 74, 01307 Dresden, Germany; 2 Working Group Focal and Micro Therapy, German Association of Urology, Berlin, Germany

**Keywords:** Focal therapy, Long-term follow-up, Hemiablation, Prostate cancer, VTP

## Abstract

**Purpose:**

Vascular-targeted photodynamic therapy (VTP) is an approved treatment option for unilateral low-risk prostate cancer (PCa).

**Methods:**

Patients with unilateral low- or intermediate-risk PCa undergoing hemiablation by VTP were evaluated in a real-world setting. Oncological outcome after VTP was measured by MRI-based re-biopsy at 12 and 24 months. Functional outcome after 1 year was investigated by IIEF-5 and IPSS questionnaires. Progression was defined as the evidence3 of ISUP ≥ 2 PCa.

**Results:**

At any control biopsy (*n* = 46) after VTP, only 37% of patients showed no evidence of PCa. Recurrence-free survival was 20 months (95% CI 4.9–45.5) and progression-free survival was 38.5 months (95% CI 33.5–43.6 months). In-field and out-field recurrent PCa occurs in 37% (55% ISUP ≥ 2 PCa) and 35% (56% ISUP ≥ 2 PCa). Seventy-nine percent of patients preserved erectile function, respectively. Ten percent of patients presented long-term bladder outlet obstruction. None of the patients presented incontinence.

**Conclusion:**

Due to the high-recurrence in- and out-field recurrence rate in a mainly low-risk prostate cancer cohort, VTP has to be regarded critically as a therapy option in these patients. Pre-interventional diagnostic evaluation is the main issue before focal therapy to reduce the risk of tumor recurrence and progression.

## Introduction

Nowadays, prostate cancer (PCa) diagnosis is preponed due to widespread PSA testing and improved PCa detection [1–3]. Moreover, due to the use of multiparametric magnetic resonance imaging (mpMRI) and targeted prostate biopsy, PCa can be localized more precisely [4, 5]. This allows the application of more precise partial ablation techniques as treatment option. Focal therapy (FT) allows to treat focal PCa and to preserve healthy tissue with a lower risk of sexual and bladder dysfunction with sufficient oncological control [3, 6, 7]. FT has become a promising alternative treatment option for low- and early intermediate-risk PCa [3, 6]. However, pre-interventional accurate diagnostic and tumor control by imaging and targeted and systematic biopsies are mandatory [8]. FT is implemented in national and international guidelines [8]. The most frequently applied FT modalities are high-intensity focused ultrasound (HIFU) and cryotherapy [1, 3, 6]. Nevertheless, the evidence for these procedures is still insufficient. Only single-center, prospective or retrospective studies currently demonstrate a therapeutic benefit in locally defined low- to early intermediate-risk PCa. So far, vascular-targeted photodynamic therapy (VTP) is the only FT modality that has shown efficacy over active surveillance in unilateral low-risk PCa in a phase III randomized-controlled trial (PCM301 trial) [9, 10].

It was demonstrated after a 4-year follow-up that patients undergoing VTP showed a lower conversion rate to radical treatment than patients undergoing AS [9, 10]. VTP was approved by the European Medical Agency (EMA) for the treatment of unilateral low-risk PCa [7, 11]. Moreover, VTP was the only FT modality which was reimbursed by the health insurances in Germany [7, 11]. However, in 2022, VTP based on the photosensitizer Padeliporfin, which is activated light of weave lengths of 753 nm [12], was stopped by the manufacturing company for the indication of unilateral low-risk PCa. Nevertheless, since the launch of VTP, our center was the first which was able to establish the procedure in the clinical setting outside of a trial and to treat 60 patients. In this paper, the long-term oncological outcome of VTP under real-world conditions will be presented.

## Patients and methods

Sixty consecutive patients with unilateral low- or early intermediate-risk PCa underwent VTP. The study was approved by the Institutional Review Board of the Technische Universität Dresden (Vote: BO-EK-259062020).

Inclusion criteria were unilateral low-risk PCa of clinical stage ≤ cT2a, ISUP 1 and PSA ≤ 10 ng/mL according to the EMA approval [7]. Three patients with intermediate-risk PCa underwent VTP. Exclusion criteria for VTP were bothersome lower urinary tract symptoms or a significant residual urine volume. Multiparametric MRI (mpMRI) prior to VTP was initially preferred but not mandatory since it was not required in the PCM301 trial [9]. However, in the further course, mpMRI was mandatory before VTP and targeted biopsies were additionally performed in the presence of PI-RADS ≥ 3 lesions.

VTP performed as hemiablation took place under general anesthesia. The detailed technique was described earlier [7, 9]. After the procedure, the Foley catheter was routinely removed on day 2. Treatment-related complications according to Clavien–Dindo. Primary oncological outcomes were recurrence-free survival for any PCa and progression-free survival defined as the evidence of ISUP ≥ 2 PCa.

Follow-up consisted of regular PSA monitoring every 3 months during the first 2 years. MpMRI and targeted biopsy in case of evidence of PI-RADS ≥ 3 lesions and a systematic biopsy covering the treated (in-field) and untreated lobe (out-field) were performed after 12 and 24 months. Tumor recurrence was defined as the evidence of any PCa. Tumor progression was defined as the evidence of ISUP ≥ 2 PCa in control biopsy.

To determine erectile function 6–12 months post-interventional, we used International Index of Erectile Function 5 (IIEF-5) questionnaire. For assessing bladder function, we used the International Prostate Symptom Score (IPSS) questionnaire.

Data analyses of quantitative variables were presented as numbers (*n*), median, mean, standard deviation (SD) and interquartile range (IQR). Qualitative variables were analyzed through frequency counts. Recurrence- and progression-free survival was evaluated by Kaplan–Meier curves. Cox regression analysis was performed to identify predictors for recurrence and progression. Comparison of means was performed by two-sided Student’s *t* test. A *p* value of < 0.05 was defined to indicate statistical significance. All statistical analyses were performed using the SPSS 27.0 (IBM corp., Armonk, NY, USA) software.

## Results

Between May 2018 and December 2022, 60 patients were scheduled for VTP. Patients’ characteristics, treatment data and oncological outcome are presented in Table 1. Fifty-seven patients fulfilled all VTP inclusion criteria. Three patients presented with an early intermediate-risk, low-volume PCa and received VTP as an individual treatment option.

**Table 1:** Description of the study cohort, intraoperative data and oncological and functional outcome after VTP

Parameter	
Age (years; median (IQR))	64 (57; 71)
iPSA (ng/ml; median (IQR))	5.3 (4.5; 7.3)
Prostate volume (mL; median (IQR))	40 (30; 50)
PSA density (ng/ml^2^; median (IQR))	0.14 (0.10; 0.21)
Number PSA density > 0.15ng/ml^2^	
Initial ISUP (*n*; %)	
ISUP 1	57
ISUP 2	3
Pre-interventional MRI (*n*; %)	
PI-RADS 2	6 (10)
PI-RADS 3	14 (23)
PI-RADS 4	26 (43)
PI-RADS 5	4 (7)
No pre-interventional MRI	10 (17)
Number of positive biopsy cores (*n*; median (IQR))	1 (1; 2)
Maximal tumor infiltration (%; median (IQR))	40 (5; 30)
Maximal tumor length (mm; median (IQR))	4 (2; 10)
IPSS preoperative	
IPSS ≤ 7 (*n*; %)	41 (68)
IPSS > 7 (*n*; %)	14 (23)
IIEF-5 preoperative	
IIEF-5 ≥ 22 (*n*; %)	29 (48)
IIEF-5 < 22 (*n*; %)	28 (61)
Treatment parameters	
Treated prostate volume (mL; median (IQR))	27 (20; 33)
Light density index (cm^−2^, median /IQR))	1.3 (1.1; 1.55)
Treatment time (min, median (IQR))	105 (90; 116)
Clavien–Dindo (*n*; %)	
I	20 (33)
II	8 (13)
≥ III	0
Post-interventional follow-up	
Follow-up (months, median (IQR)	21 (12–33)
Patients with 12-month biopsy (*n*; %)	46 (77)
Patients with 24-month biopsy (*n*; %)	15 (25)
Patients lost to follow-up	4 (7)
Any post-interventional MRI (*n* = 65)	
In-field	
Tumor suspicious	10 (15)
Not tumor suspicious	55 (85)
Out-field	
PI-RADS 2	42 (65)
PI-RADS 3	12 (18)
PI-RADS 4	11 (17)
PI-RADS 5	0 (0)
Oncological follow-up	
Recurrent prostate cancer (*n*; %) (46 patients)	26 (57)
ISUP 1	12 (26)
ISUP ≥ 2	14 (30)
Recurrent prostate cancer—(*n*; %) (46 patients)	
In-field	18 (39)
ISUP 1	8 (17)
ISUP 2	10 (22)
ISUP ≥ 3	0 (0)
Out-field	16 (35)
ISUP 1	7 (15)
ISUP 2	7 (15)
ISUP ≥ 3	2 (5)
Both (max. ISUP)	6 (13)
ISUP 1	0 (0)
ISUP 2	5 (11)
ISUP ≥ 3	1 (2)
Unknown localization	1 (2)
IPSS post-interventional	
IPSS ≤ 7 (*n*; %)	33 (55)
IPSS > 7 (*n*; %)	8 (13)
IIEF-5 post-interventional	
IIEF-5 ≥ 22 (*n*; %)	17 (28)
IIEF-5 < 22 (*n*; %)	18 (30)
Residual volume post-interventional > 50mL (*n*; %)	6 (10)

*iPSA*, initial PSA; *SD*, standard deviation

**Fig. 1:**
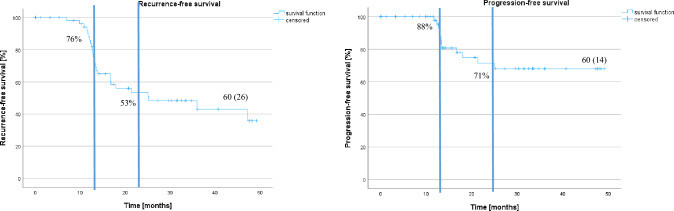
Recurrence-free survival for any PCa (**A**) and clinically significant PCa (ISUP ≥ 2) (**B**)

Four patients were lost to follow-up since they evaded further follow-up. Seventy-seven percent of patients (*n* = 46) underwent at least one follow-up mpMRI and control biopsy. Fifteen patients (25%) and three patients (5%) underwent a second and further control biopsy, respectively. The mean PSA value decreased after 6 and 12 months from initial 6.1 ng/mL (standard deviation (SD) ± 3.0 ng/mL; *n* = 60) to 3.9 ng/mL (SD ± 2.4; *n* = 42; *p* < 0.001) and to 4.4 ng/mL (SD ± 3; *n* = 45; *p* = 0.001), respectively, and remained stable. Fifty percent patients (*n* = 30) showed a PSA decline ≥ 50% after 6 months. After a median follow-up of 21 months (inter quartile range (IQR) 12–33 months), 63% of patients (29/46) presented PCa recurrence and 33% patients (15/46) showed PCa progression. Median recurrence-free survival for any cancer was 20 months (95% CI 4.9–45.5 months) and mean progression-free survival was 38.5 months (95% CI 33.5–43.6 months) (Fig. 1). The median progression-free survival has not been reached yet. In Cox regression analysis, no clinical parameter could be determined as predictor for PCa recurrence or progression.

In first control biopsy, 48% of the patients (22/46) showed evidence of PCa (ISUP 1: *n* = 10; ISUP ≥ 2: *n* = 12). In the second control biopsy, PCa was proven in 40% (6/15) (ISUP 1: *n* = 3; ISUP ≥ 2: *n* = 3). In further control biopsies, one patient presented low-risk PCa. All control biopsies together, in-field and out-field tumor recurrence was detected in 37% (18/46) (ISUP 6: *n* = 8; ISUP ≥ 2: *n* = 10) and 35% (16/46) of patients (GS6: *n* = 7; ISUP ≥ 2: *n* = 9), respectively. Thirteen percent of patients (6/46) presented PCa in- and out-field (ISUP 1: *n* = 4; ISUP ≥ 2: *n* = 2).

Fifteen percent patients (= 10) showed evidence of tumor recurrence in mpMRI (in total 65 follow-up mpMRI) of the treated lobe. In these cases, in-field PCa was found in seven cases. On the untreated lobe, 21 patients showed PI-RADS ≥ 3 lesions.

Perioperative complications and functional outcomes are presented in Table 1. There was no complication with Clavien–Dindo ≥ 3 within 90 days after the intervention. The most common complications were: hematuria (10%) symptoms of urge and urge incontinence (15%) which were resolved within 6–8 weeks, residual urine (10%), urinary retention (7%) and urinary tract infection/fever (10%). 6–12 months after treatment, 79% of patients with preoperative IIEF ≥ 22 retained erectile function.

In case of recurrence of ISUP 1 PCa, nine patients followed active surveillance, and one patient underwent VTP of the other lobe. Eleven and four patients underwent salvage radical prostatectomy or salvage radiotherapy due to tumor progression. Final pathology in case of salvage RPx performed at the University Hospital Dresden (*n* = 7) revealed organ-confined disease in six patients and extracapsular tumor extent in one patient. One patient presented lymph node metastasis. All patients presented ISUP 2 PCa and R0 resection status. In none of the patients, nerve sparing could be performed on the pretreated side.

## Discussion

Although VTP was the only FT modality which has presented level-one evidence [8] and the treatment was reimbursed by some health systems according to the EMA approval [7, 9], the manufacturing company stopped VTP for the indication of unilateral low-risk PCa in the end of 2022. Our clinic was the first which implemented VTP outside a clinical trial. Since the implementation in May 2018, we have treated 60 patients until the end of 2022. In this retrospective study, we present the long-term follow-up VTP treatment in a real-world setting.

Herein, we could show that VTP is a feasible and safe treatment. The most common side effects were prolonged hematuria, urge and urine retention. With regard to functional outcomes, 79% of patients with pre-VTP IIEF-5 ≥ 22 retained erectile function and 75% had no bladder outlet obstruction. Especially regarding the preservation of erectile function, VTP and other FT modalities offer benefit [13].

Until now, only the German S3 guideline presents stringent recommendation for diagnostic and follow-up pathways in patients undergoing FT including imaging and control biopsies. Beside PSA testing, follow-up mpMRI and biopsies covering the in-field and out-field region have been recommended [3, 8, 14, 15]. However, stringent recommendations for a structured follow-up after FT are still missing [8, 16]. Regarding the follow-up after FT, data for the interpretation of PSA course and biochemical recurrence are still pending [17, 18]. Moreover, especially the PSA course depends on the ablation volume. In our study, we observed a PSA decrease after FT which remained stable during the follow-up. Another important aspect, mpMRI, after FT is recommended, although exact criteria for interpretation of post-FT alterations in mpMRI have not been yet established. Giganti et al. proposed a new scoring system, the Prostate Imaging after Focal Ablation (PI-FAB), to evaluate according to contrast-enhanced, diffusion weighted and T2 sequences compared to the pre-interventional mpMRI [19]. However, the evaluation of this score is still pending.

Within a median follow-up of 21 months, in 46 patients undergoing control biopsy, only 37% of patients did not show evidence of tumor recurrence. Thirty-three percent of patients showed tumor progression. Compared to the data we published in 2021, we recognize an increase in the portion of patients with tumor recurrence and progression. Herein, we had presented the 1-year follow-up cohort (median follow-up of 12.3 months) demonstrating a recurrence and progression rate of 55% and 27%, respectively [7].

This number is considerable but comparable to the 2-year follow-up data of the PCM301 trial demonstrating a progression rate of 28% in the VTP arm [9]. Regarding the high progression rate of the active surveillance (58%) in the PCM301 trial, VTP seems to be a valuable option in the treatment for low-risk PCa [9]. However, compared to data of other active surveillance cohorts [20, 21]. With low-risk PCa, the progression rate seems to be high, especially regarding a relatively high ablation volume of 27 mL in the sense of a hemiablation. Moreover, in the 4-year follow-up of the PCM301 trial the whole-gland and in-field progression rate was approximately 25% and 19% after 24 months [10].

It has to be mentioned that in the upfront but also in the follow-up diagnostic of the PCM301 trial, imaging with MRI, targeted biopsy or saturation biopsies were not mandatory [9]. Therefore, under-grading and -staging of the tumor might be very probable.

Compared to other FT modalities as HIFU, IRE or cryoablation with a detection rate of csPCa in the treated lobe of 14, 8.5 and 10%, respectively [6], our cohort present a relatively high in-field detection rate of 39%.

Westhoff et al. showed in the recently published FOXPRO trial investigating HIFU treatment in 50 patients with low- (54%) to intermediate-risk (46%) PCa, an overall recurrence rate of 40% and a detection rate of 32% (ISUP ≥ 2 16%) in the treated region [22]. However, other retrospective real-world single-center studies demonstrate high PCa recurrence rate up to 44% after one year [23]. Therefore, the high-recurrence rate under real-world conditions in FT has to be considered and reflects the high likelihood of under-detection and under-grading by initial diagnosis [4, 5].

In our study, over 17% of patients did not undergo pre-VTP mpMRI, which might be a risk of tumor misclassification in these patients. Interestingly, post-VTP mpMRI revealed in-field tumor suspicious in only 15% of patients, whereas 32% presented PI-RADS ≥ 3 out-field. However, the rate of tumor progression was comparable in the in- and out-field region. Especially in-field tumor recurrence and progression might represent an under-treatment. However, since it is not possible to define exactly the borders of the ablation zone of the treated lobe, the differentiation of tumor recurrence within or in the neighboring region is not feasible in prostate biopsy. Noweski et al. observed in 50% of VTP patients a positive follow-up biopsy whereby 50% of detected PCa were located in the treated lobe [24]. Overall, 15% out of this cohort presented PCa progression to ≥ ISUP 2 [24]. The higher recurrence rate in our cohort might be caused due to the stringent use of mpMRI in the diagnostic and follow-up pathway. All patients were diagnosed at our center or, if otherwise, they received confirmation biopsy. However, 17% of them did not receive mpMRI before which might explain underdiagnosis and lead to a higher recurrence- and progression rate. Most of the follow-up biopsies were performed in-house after mpMRI.

Patients considering VTP as treatment option should be aware of this considerable progression rate necessitating radical treatment. In our study, seven and four VTP patients underwent salvage RPx and radiotherapy due to tumor progression, respectively. In case of RPx, a R0 resection status was achieved in all patients. In none of the patients, nerve-sparing surgery at the treated lobe was performed. However, one patient presented a locally advanced PCa with evidence of lymph node metastases. Salvage RP is described as oncologically and functionally safe with acceptable side effects [25–27]. However, that after VTP only in one-third of patients a bilateral nerve sparing could be performed. Moreover, post-VTP RP was associated with positive surgical margins on the previously treated side [26].

Our study has some limitations. First, this is a retrospective study with a potential recall bias. It has to be mentioned that outcome was documented in 92% and only 77% of patients underwent control biopsy due to the fact that the last treated patients presented a too short follow-up. Moreover, 6% were lost to follow-up. Thus, further studies are required to confirm low complication rate of VTP and the oncological and functional outcome. Secondly, only 83% of patients underwent pre-VTP mpMRI and targeted biopsy which may lead to a detection bias, therefore, to a higher recurrence and progression rate. Furthermore, we could not distinguish in follow-up biopsy if cores with evidence of PCa were located within the treated regions or next to it. Another limiting factor is the relatively small size of the study population group.

## Conclusion

Due to the high-recurrence in- and out-field recurrence rate in a mainly low-risk prostate cancer cohort, VTP has to be regarded critically as a therapy option in these patients. Pre-interventional diagnostic evaluation is the main issue before focal therapy to reduce the risk of tumor recurrence and progression.
